# How to Integrate Surgery into the Multidisciplinary Treatment of Liver-Only Metastatic Colorectal Cancer

**DOI:** 10.3390/cancers18030489

**Published:** 2026-02-02

**Authors:** Leticia Pérez-Santiago, Dixie Huntley Pascual, José Saúl Sánchez Lara, Marisol Huerta, Dimitri Dorcaratto

**Affiliations:** 1 Colorectal Surgery Unit, Department of General and Digestive Surgery, Hospital Clínico Universitario, INCLIVA Biomedical Research Institute, 46010 Valencia, Spain; letperezsantiago@gmail.com; 2 Department of General and Digestive Surgery, Hospital Clínico Universitario, INCLIVA Biomedical Research Institute, 46010 Valencia, Spain; escorial730@yahoo.com (D.H.P.); saullara92@hotmail.com (J.S.S.L.); 3 Department of Medical Oncology, Hospital Clínico Universitario, INCLIVA Biomedical Research Institute, University of Valencia, 46010 Valencia, Spain; huerta.mha@gmail.com; 4 Hepatobiliopancreatic Surgery Unit, Department of General and Digestive Surgery, Hospital Clínico Universitario, INCLIVA Biomedical Research Institute, 46010 Valencia, Spain

**Keywords:** colorectal liver metastases, multidisciplinary teams, individualized therapy, hepatectomy, conversion therapy, tumor biology, future liver remnant

## Abstract

**Background:** Colorectal liver metastases (CRLM) represent a major determinant of prognosis in patients with metastatic colorectal cancer and account for a substantial proportion of cancer-related mortality worldwide. Over the last decades, survival outcomes have improved significantly as a result of advances in systemic therapies, refinement of surgical techniques, and, most importantly, the widespread implementation of multidisciplinary management strategies. Within this evolving landscape, surgery remains the cornerstone of potentially curative treatment, although its optimal integration with systemic and locoregional therapies requires careful patient selection and individualized treatment planning. **Methods:** This narrative review explores the contemporary role of surgery within the multidisciplinary management of CRLM, emphasizing how surgical decision-making is integrated with medical oncology, radiology, interventional procedures, and emerging technologies. **Results:** The pivotal role of multidisciplinary team meetings in defining resectability, treatment sequencing, and therapeutic intent is highlighted. Key technical and oncological criteria guiding upfront resection, neoadjuvant or conversion strategies, and staged approaches are reviewed, including assessment of future liver remnant, optimization of liver volume and function, tumor burden, molecular profile, and dynamic prognostic models. In addition, the review summarizes current evidence supporting parenchyma-sparing liver surgery and the integration of local therapies such as thermal ablation, irreversible electroporation, stereotactic body radiotherapy, selective internal radiation therapy, and hepatic artery infusion chemotherapy within multimodal treatment algorithms. Complex clinical scenarios, including synchronous disease, extensive bilobar metastases, chemotherapy-associated liver injury, and the emerging role of liver transplantation in highly selected patients with liver-only disease, are also addressed. **Conclusions:** Modern CRLM management has evolved toward a highly individualized, biology-driven approach in which surgery is optimally integrated within a multidisciplinary framework to maximize curative potential and long-term survival.

## Simple Summary

Colorectal liver metastases (CRLM) represent a major determinant of prognosis in metastatic colorectal cancer. Despite an improvement in survival as a result of advances in systemic therapy, surgical techniques, and multidisciplinary management, the optimal integration of surgery with systemic and locoregional treatments remains an area of ongoing debate. This narrative review is proposed to clarify the contemporary role of surgery within a multidisciplinary framework for CRLM. Specifically, the review aims to delineate the technical, oncological, and biological factors that guide resectability assessment, treatment sequencing, and therapeutic intent, including upfront resection, neoadjuvant or conversion strategies, and staged approaches. By addressing parenchyma-sparing surgery, locoregional therapies, and challenging clinical scenarios, this review seeks to provide a coherent, practice-oriented perspective. The findings are expected to support more consistent, individualized, and biology-driven decision-making, thereby informing multidisciplinary discussions and contributing to improved patient selection and long-term outcomes in CRLM.

## 1. Introduction

Colorectal cancer is the third most common cancer and the second leading cause of cancer-related mortality worldwide [1]. Approximately 25–30% of these patients will develop liver metastases during the course of their disease, either synchronously or metachronously [2,3]. Over recent years, overall survival (OS) in this group of patients has increased, largely due to a more collaborative approach that has improved patient selection, allowed better definition of therapeutic sequencing, and facilitated more precise treatment planning [4,5,6].

It is now well established that multidisciplinary management improves survival outcomes for patients with colorectal liver metastases (CRLM) and hepatocellular carcinoma, with surgery representing a central pillar of treatment [7,8]. In this context, this review examines the contemporary role of surgery within the multidisciplinary management of CRLM, emphasizing its integration with systemic and locoregional strategies to maximize curative potential and long-term survival.

## 2. Multidisciplinary Team Meetings in Contemporary Cancer Care

The multidisciplinary approach to cancer patients has been described since the 1970s [9]. However, the concept of today’s multidisciplinary teams (MDTs) emerged around the 1990s, when these meetings began to bring together specialists who regularly convened to discuss complex cases and reach treatment decisions collectively. Nowadays, MDT meetings (MDTMs) are a fundamental element in the management of almost all cancers, including CRLM [10].

Building on this historical evolution, the clinical importance of MDTMs is now clearly recognized. Patients diagnosed with CRLM are complex and require coordinated, joint management by an MDT. Team members must choose among the always rapidly evolving therapeutic options to achieve the best possible oncologic outcomes [11]. Different studies show that access to MDT evaluation optimizes treatment strategy, improves adherence to clinical guidelines [12], speeds up diagnosis and initiation of treatment, and enhances both survival and patient satisfaction [13,14]. In a systematic review by Kočo et al., an increase in OS was observed among patients with colorectal, lung, prostate, or breast cancer who were evaluated by an MDT [15].

### 2.1. Composition of MDTs and the Role of the Surgeon

As mentioned earlier, MDTs are periodic meetings in which clinical cases are discussed, and their composition depends on the pathology being addressed [16,17]. In the specific case of CRLM, the team should consist of liver surgeons, colorectal surgeons, hepatologists, gastroenterologists, endoscopists, medical oncologists, radiologists, interventional radiologists, radiation oncologists, pathologists, cancer nurse specialists, and stomatherapy professionals [10,18,19,20,21]. Evidence shows that patients with CRLM assessed in specialized liver MDTs achieve superior disease-free and overall survival [11,22].

Within this multidisciplinary framework, the role of the surgeon—particularly the hepatobiliary (HPB) surgeon—is of great importance. Teams that include HPB surgeons achieve higher resection rates and better survival [22]. Almlöv et al. found that MDT evaluation, including a liver surgeon, was independently associated with improved survival in patients with CRLM [23].

### 2.2. Role of MDTs in the Management of CRLM

The central role of the MDT is to optimize diagnosis and determine the best therapeutic strategy, considering all available options to achieve the best oncologic outcome [20]. The MDT must coordinate the overall assessment of a patient diagnosed with CRLM. Its responsibilities can be divided into the following points: (1) evaluation of the patient’s clinical condition and initial performance status; (2) assessment of resectability and/or the need for additional liver-directed therapies based on imaging studies; (3) evaluation of the feasibility and safety of hepatic resection (surgical criteria); and (4) assessment of tumor biology and prognostic factors [18].

Once these points have been evaluated and discussed, the MDT determines the best therapeutic option for each patient. Specifically, it identifies candidates for upfront resection, determines which patients require preoperative chemotherapy, evaluates the need for adjunctive interventions such as ablative therapies or liver-volume optimization techniques, and establishes the optimal sequencing of treatment modalities [24]. In this way, MDTMs provide a structured framework to individualize treatment plans for patients with CRLM.

#### Assessment of Initial Clinical Status and Performance Status

The patient’s overall clinical condition, comorbidities, and performance status are fundamental elements that MDT members must consider when determining therapeutic strategies. The most widely used scales to evaluate performance status are the Eastern Cooperative Oncology Group (ECOG) score and the Karnofsky Performance Status (KPS) score [25,26].

In addition to performance status, a comprehensive preoperative assessment is crucial. The patient should undergo a complete medical history, physical examination, cardiopulmonary evaluation, and anesthetic assessment. There is increasing interest in the role of nutritional assessment in these patients, as interventions that improve nutritional status prior to treatment may lead to better outcomes in patients with CRLM [27]. Together, these evaluations help the MDT define which patients are suitable candidates for major liver surgery or other invasive therapies.

### 2.3. Gap Between Eligible Candidates for Surgery and Effectively Resected Patients

Despite the structured and comprehensive role of MDTMs in CRLM management, several observational studies have consistently reported a substantial gap between patients considered theoretically eligible for curative-intent surgery and those who ultimately undergo hepatic resection. While part of this discrepancy may be related to the absence of universally accepted resectability criteria and to interobserver variability, growing evidence indicates that organizational factors play a major role. In particular, the lack of hepatobiliary surgical expertise within MDTs has been associated with underestimation of resectability and inappropriate allocation of patients to non-surgical or palliative treatment strategies [13]. Studies evaluating dedicated hepatobiliary MDTs have shown higher resection rates and improved survival outcomes compared with non-specialized multidisciplinary panels, highlighting the critical impact of specialist surgical input on treatment allocation and long-term outcomes [22]. These findings support the concept that a proportion of so-called “failure-to-cure” cases may be attributable not only to adverse tumor biology or technical constraints, but also to suboptimal multidisciplinary evaluation [28]. Consequently, ensuring access to experienced hepatobiliary surgeons within MDTs appears essential to reduce the gap between surgical eligibility and effective resection in patients with CRLM.

## 3. Imaging for Determining Resectability and Preoperative Planning

MDTs rely on high-quality imaging—interpreted in collaboration with specialized radiologists—to determine disease staging, tumor burden, hepatic lesion distribution, and the presence of extrahepatic disease (EHD), all of which are essential for defining the surgical plan [29]. Thus, imaging plays a pivotal role in bridging diagnostic information with surgical decision-making.

**Figure 1:**
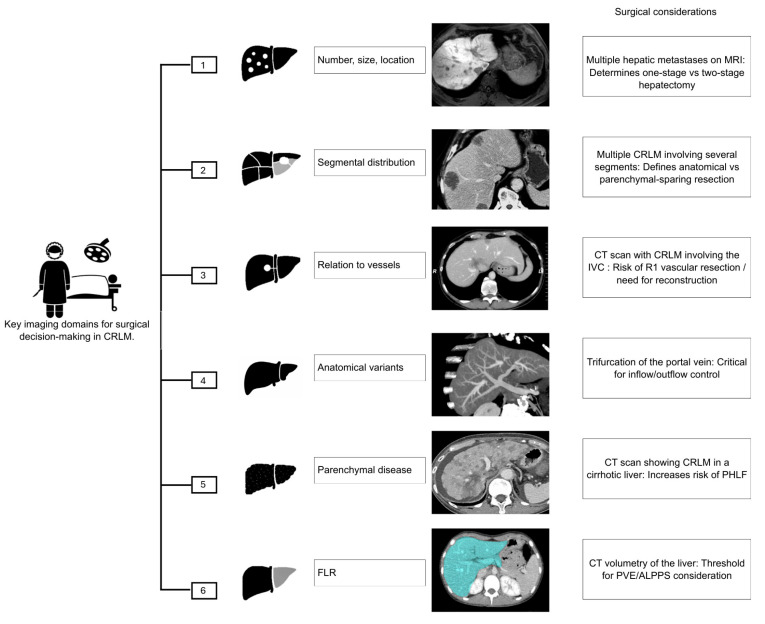
Key imaging domains guiding surgical decision-making in patients with CRLM. CRLM: Colorectal Liver Metastases; MRI: Magnetic Resonance Imaging; CT: Computed Tomography; IVC: Inferior Vena Cava; R1: Microscopically positive resection margin; PHLF: Post-Hepatectomy Liver Failure; FLR: Future Liver Remnant; PVE: Portal Vein Embolization; ALPPS: Associating Liver Partition and Portal vein Ligation for Staged Hepatectomy.

The primary objective of imaging in CRLM is to provide the most accurate roadmap for assessing resectability [29]. In patients in whom metastasis is suspected, magnetic resonance imaging (MRI) is the imaging test of choice, especially for subcentimeter lesions [30]. When it is determined that the lesion is confined to the liver, surgical resection offers the best long-term survival. To achieve this, proper surgical planning and a thorough evaluation of resectability are necessary. Specifically, the surgeon needs to know (1) the number, size, and location of the lesions; (2) their segmental distribution; (3) their relationship to the liver capsule, hepatic hilum, and major vascular and biliary structures; (4) the presence of anatomical variants; (5) any underlying parenchymal liver disease; and (6) when appropriate, an assessment of the functional liver remnant (FLR) [29] (Figure 1).

Certain radiologic patterns may preclude an upfront curative resection. These “game-changing” lesions include deep-seated metastases involving the hepatic venous confluence or inferior vena cava; multiple lesions closely associated with both right and left portal pedicles, all three hepatic veins, or the hepatic hilum; numerous small, disseminated metastases; and caudate lobe lesions [31,32]. Identifying these patterns early allows the MDT to consider alternative strategies such as conversion chemotherapy or staged procedures.

Recent technological developments have significantly improved the precision of hepatic surgery. Three-dimensional reconstruction and virtual surgical planning allow detailed evaluation of liver volume, FLR, tumor boundaries, and spatial relationships with hepatic vasculature [33]. These tools may reduce the risk of complications, lower recurrence rates, and help avoid unnecessary major hepatectomies by enabling more accurate, parenchyma-sparing approaches [34,35,36]. Consequently, modern imaging not only informs whether surgery is possible, but also refines how it should be performed.

In borderline resectable disease, imaging also plays a critical role in identifying candidates for neoadjuvant chemotherapy to increase the likelihood of achieving an R0 resection. This dynamic reassessment of resectability over time exemplifies the iterative interaction between systemic therapy, imaging, and surgical planning in MDT practice.

Imaging remains essential across all phases of CRLM management—detection, staging, therapy planning, and treatment response evaluation [37,38]. Its utility now extends to novel applications such as radiomics, machine learning, and artificial intelligence, which are increasingly investigated for individualized therapeutic planning. To better understand their specific contributions, each imaging modality is summarized below.

### 3.1. Contrast-Enhanced Computed Tomography (CT)

A contrast-enhanced computed tomography (CT) scan of the chest, abdomen, and pelvis is the imaging test most commonly used for the detection and initial staging of CRLM [20]. Its sensitivity and specificity for detecting CRLM range from 51.8 to 84.6% and 77 to 98%, respectively [39]. It is the preferred modality during the initial workup because it is cost-effective and widely available. However, in patients already known to have CRLM, MRI should be subsequently performed, especially in those who have received neoadjuvant chemotherapy, since CT sensitivity decreases to around 65% after chemotherapy [40].

A meta-analysis showed that in patients treated with neoadjuvant chemotherapy, CT sensitivity for detecting CRLM was 69.9%, compared to 85.7% for MRI [41]. These findings reinforce the complementary role of CT and MRI in accurately characterizing liver disease before surgery.

### 3.2. Liver MRI

Liver MRI with hepatocyte-specific contrast is recommended in the evaluation of CRLM because it offers superior sensitivity and specificity, especially for small hepatic lesions that may go unnoticed on CT [42,43], and in those patients who have already received neoadjuvant chemotherapy [41]. As such, MRI has become the key modality for refining surgical planning in many centers.

The value of hepatocyte-specific MRI in optimizing CRLM management was demonstrated in the CAMINO study, which assessed the clinical impact of adding MRI to CT in patients scheduled for local therapy based on CT findings alone. The study showed that MRI led to changes in the local treatment plan in 31% of patients [44]. This underscores the importance of advanced MRI protocols within MDT-driven decision-making.

### 3.3. Positron Emission Tomography PET/CT and PET/MRI

Positron emission tomography combined with CT (PET/CT) or MRI (PET/MRI) complements CT and MRI by helping detect occult extrahepatic metastases that may alter the initial therapeutic plan [45,46]. The European Society for Medical Oncology (ESMO) clinical guidelines recommend PET/CT in patients with rising tumor markers but no clear evidence of metastatic disease, or to define the extent of metastatic involvement in potentially resectable cases [20].

PET/MRI has recently emerged as a newer imaging tool, although it is still not widely used due to its high cost and limited availability. There are limited data, but in the studies comparing PET/MRI with PET/CT, PET/MRI has shown better performance for evaluating CRLM and has led to changes in therapeutic management in a significant proportion of patients [47]. These developments suggest that hybrid imaging may further refine staging and treatment selection in selected patients.

### 3.4. Intraoperative Imaging

Despite all the imaging performed during the preoperative evaluation, it is still necessary during surgery for liver metastasis resection to carry out a thorough intraoperative exploration. This includes palpation of the liver, visual inspection, and the use of intraoperative ultrasound (IOUS) or contrast-enhanced intraoperative ultrasound (CE-IOUS) to confirm the number and size of metastases and to identify any lesions that may have gone unnoticed on preoperative imaging [48].

IOUS has very high sensitivity and specificity—94–100% and 95–100%, respectively—for determining the size and location of hepatic metastases. It also helps identify blood vessels and biliary structures during surgery, which has a positive impact on resection margins and overall surgical outcomes [49,50,51]. Therefore, IOUS is now considered a standard adjunct in oncologic liver surgery.

CE-IOUS uses a microbubble-based contrast agent that provides real-time information about CRLM and their vascularity. This gives CE-IOUS an advantage over conventional IOUS, offering higher sensitivity and specificity [52]. At present, CE-IOUS is considered the best method for intraoperative evaluation of CRLM, and its use is recommended during hepatic resections due to the valuable information it provides to the surgeon [52,53]. Arita et al. highlighted the value of CE-IOUS in detecting disappearing liver metastases (DLM) after chemotherapy, identifying more lesions than conventional IOUS [54]. Their findings show that CE-IOUS is an essential tool for optimizing curative resection in patients with CRLM and DLM [55].

## 4. Surgical and Oncological Criteria Used to Determine Whether a Patient Should Initially Undergo Systemic Therapy or Surgical Intervention

In the last 50 years, advances in surgical techniques and contemporary systemic chemotherapy regimens have increased the overall survival (OS) for patients with colorectal liver metastases (CRLM) undergoing hepatic resection [56,57]. The challenge, however, remains in selecting the most appropriate and personalized treatment strategy for each patient.

### 4.1. Surgical Factors (Technical)

All patients with CRLM who have adequate physiological reserves and performance status should be considered for hepatic resection, the standard treatment for resectable disease [20].

#### 4.1.1. Technical Feasibility

Innovative approaches in surgery and an increased understanding of liver anatomy and physiology have shifted the boundaries of unresectable disease and made tumor resection safer. However, complete resection should be pursued only when anatomically feasible, according to the extent of disease.

Resectability of CRLM has been defined by the European Society for Medical Oncology (ESMO) as a margin-negative (R0) resection where the future liver remnant is adequate to maintain sufficient hepatic function [20].

Preoperative imaging, as described previously, is essential in determining whether CRLMs are resectable. In addition, intraoperative ultrasound (IOUS) also plays a key role in confirming the number, size and location of liver lesions and detecting metastases potentially missed by preoperative images [48,58].

#### 4.1.2. Resection Margins

Traditionally, the standard resection margin has been >10 mm [59]. However, several large retrospective studies have evaluated margin status and found that a negative margin of <10 mm is not associated with reduced survival. For example, Sadot et al. published a study of 4915 patients who underwent resection for CRLM at the Memorial Sloan Kettering Cancer Institute, in which they compared various margin widths, including submillimeter margins and found there was no statistically significant difference in survival between the 1 to 9 mm and >10 mm groups [60]. These data suggest that wider surgical margins are preferable; however, a narrow but negative margin should not preclude resection.

Beyond absolute margin width, the anatomical relationship between CRLMs and major intrahepatic vessels is increasingly recognized as a critical determinant of resectability. R1 vascular resection describes the detachment of CRLMs from major intrahepatic vessels in the absence of true vascular wall infiltration. Recent evidence indicates that R1 vascular detachment can be achieved with excellent oncological outcomes [61,62,63], although further studies are warranted.

#### 4.1.3. Future Liver Remnant (FLR)

Deeply situated CRLM in proximity to major vascular or biliary structures may require major hepatic resection to achieve R0 margins. In order to prevent postoperative liver failure, preoperative evaluation of the future liver remnant and its functional adequacy is critical.

The recommended FLR depends on the quality of the remaining tissue and its ability to regenerate. For instance, up to 75% of a non-cirrhotic liver can be safely removed, while FLR should represent at least 30–35% of the total liver volume after neoadjuvant chemotherapy and approximately 40% in patients with cirrhosis. Liver volume is measured using CT/MRI volumetry, manual or software-assisted, depending on local availability [64]. Results are considered comparable when the same imaging modality and segmentation protocol are applied consistently for both pre- and post-embolization assessments.

Several strategies have been developed to augment FLR in patients whose preoperative evaluation demonstrates insufficient residual volume for a safe resection. The different approaches and their main characteristics are described in Table 1 [4,65,66]. A more detailed explanation of these strategies, with emphasis on the two-stage hepatectomy, is provided later in this manuscript.

In addition to FLR volume, hepatic function should also be evaluated to predict postoperative liver failure. The most reliable tests for assessing liver parenchyma functionality are the indocyanine green (ICG) clearance test, the hepatobiliary scintigraphy with Technetium-99 m (99 mTc HBS) and the LiMAx^®^ (Liver Maximum Capacity) test [67,68,69]. The ICG clearance test reflects hepatic blood flow and hepatocellular function, being the most widely used assay. The 99 mTc HBS is increasingly used to complement or replace ICG testing and provides segmental assessment of liver function. Finally, the LiMax^®^ measures real-time metabolic capacity via ^13^C-methacetin breath test while offering high predictive value for postoperative liver dysfunction.

### 4.2. Oncological Considerations (Prognostic)

Oncological criteria comprise prognostic factors that critically influence disease-free survival (DFS) and the potential for curative outcome. In agreement with the main clinical risk scores [70,71,72] that are currently in use to predict prognosis after liver resection, these factors include:

#### 4.2.1. Primary Tumor Characteristics: Lymph Node Involvement, Tumor Differentiation, and Tumor Location

It is well established that primary tumor lymph node positivity is an important prognostic factor in the management of CRLM. Accordingly, it is included in most clinical risk scores used to predict prognosis after liver resection [71,72,73].

Tumor differentiation should also be considered when selecting candidates for neoadjuvant chemotherapy prior to surgical resection. In fact, in an analysis of the National Cancer Database, Turner et al. reported that poorly differentiated tumors were associated with inferior overall survival among patients with colorectal liver metastases undergoing metastasectomy, supporting their classification as an adverse prognostic factor [74]. Another histological feature that has been referred to as a novel and reliable factor for predicting prognosis in CRLM is tumor grading vía poorly differentiated cluster (PDC) quantification. A PDC is a solid cancer nest lacking gland formation and comprising ≥5 cancer cells and is reportedly a prognostic parameter independent of the anatomic extent of disease [75].

A relatively recent focus in the study of prognostic factors is the location of the primary tumor, as each site tends to exhibit distinct molecular biology. In fact, it is increasingly recognized that right-sided tumors are associated with poorer prognosis compared to left-sided ones. Various studies have also found that in patients undergoing resection of colorectal liver metastases, DFS and overall survival (OS) are comparable between right-sided and rectal primaries, and that these (rectal primaries) exhibit worse outcomes than left-sided cancers, supporting the distinction between these two locations when assessing risk factors [76,77,78].

#### 4.2.2. Hepatic Tumor Burden, Localization of the Liver Lesions, and Perihepatic Lymph Node Involvement

Tumor burden, reflected by the number and size of liver metastases, remains a critical determinant of prognosis following hepatic resection. A limited number of metastases [1,22] and tumor size below 5 cm are associated with improved survival, while multiple lesions (≥4) or large tumors (>5 cm) denote more aggressive disease and poorer oncologic outcomes [59]. Despite advances in surgical and systemic therapy, these parameters continue to play a central role in risk stratification models. However, today the number of CRLM does not determine the resectability of liver disease.

Furthermore, while CRLM’s proximity to the liver capsule, the hepatic hilum or critical vascular or biliary structures can determine resectability and consequently justify the use of neoadjuvant systemic therapy, it remains an underrepresented variable in modern risk scores [79].

Perihepatic lymph node involvement reflects aggressive disease behavior, although prognosis remains more favorable when involvement is restricted to the porta hepatis nodes compared with the common hepatic artery or paraaortic regions. In carefully selected patients without celiac axis metastases, hepatic pedicle lymphadenectomy may enhance 3-year survival [73,80].

#### 4.2.3. Disease-Free Interval (Timing of Onset)

Timing of liver metastasis onset in colorectal cancer has also been established as a prognostic factor. According to a multi-societal European consensus on terminology, diagnosis and management of patients with colorectal liver metastases of 2023, liver metastases that are detected at the time of diagnosis of the primary colorectal tumor, including those detected intra-operatively, are known as synchronous metastases. Conversely, metachronous disease refers to those liver metastases that have been ruled out following cross-sectional imaging at the time of primary tumor diagnosis and are generally associated with a more favorable prognosis [81]. Metachronous metastases can be further classified as early metachronous metastases (detected up to 12 months after primary tumor diagnosis) or late metachronous metastases (more than 12 months after primary tumor detection) [81].

#### 4.2.4. Extrahepatic Metastases

Although traditionally regarded as a contraindication, extrahepatic disease no longer precludes hepatic resection in carefully selected patients when complete (margin-negative) resection or curative-intent local treatment of both intrahepatic and extrahepatic disease is feasible [82]. Nevertheless, treatment strategies in these complex cases should always be discussed within an MDT.

#### 4.2.5. Peripheral Blood Biomarkers: Carcinoembryonic Antigen (CEA) and Circulating Tumor DNA (ctDNA)

An increase in postoperative CEA levels is associated with early recurrence and may serve as a dynamic measure of tumor behavior. Historically, clinical risk scores applied varying CEA cutoffs to define prognostic risk, whereas contemporary models increasingly use CEA as a continuous variable to enhance predictive precision [83].

Another potential biomarker is ctDNA. Emerging evidence suggests that it may predict minimal residual disease and early relapse and could be useful for adjuvant therapy stratification. However, its clinical role remains investigational, and further studies are warranted [84].

#### 4.2.6. Molecular Prognostic Factors

Although the integration of genetic biomarkers into clinical decision-making is likely to personalize surgical management, refining the selection of strategies and optimizing both the timing and nature of the intervention, most are not established prognostic markers but rather have predictive value for response to selected therapies and will be briefly discussed further below [20,85].

Among these genetic biomarkers, BRAF V600 is the only one with clearly established prognostic relevance and is linked to a markedly unfavorable prognosis [20]. Although, especially in these mutations, the information provided by molecular testing may dissuade surgery if the risks from a resection exceed the benefit, it is difficult to justify denying a patient an attempted resection based solely on their mutation status. A few studies have shown that some patients are able to achieve long-term survival and may be cured despite possessing poor tumor biology [85].

The development of dynamic models integrating longitudinal laboratory markers, together with web-based tools enabling their clinical application in patients with CRLM undergoing simultaneous resection, has demonstrated promising performance and may support more precise, individualized decision-making in CRLM management [86].

In addition, artificial intelligence may support CRLM management by predicting treatment outcomes and prognosis—including recurrence and survival—thereby guiding patient selection and optimizing resource use [87].

### 4.3. Applying Surgical and Oncological Criteria to Create an Algorithm

**Figure 2:**
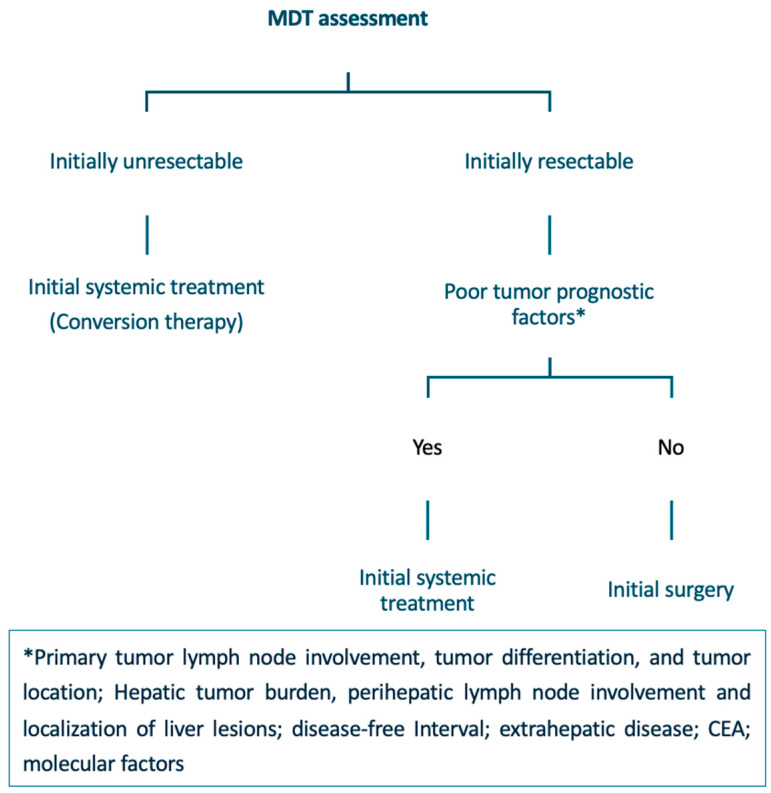
Multidisciplinary treatment algorithm for patients with CRLM. CRLM: Colorectal Liver Metastases; MDT: Multidisciplinary Team; CEA: Carcinoembryonic Antigen.

A proposed algorithm for the multidisciplinary treatment of CRLM is detailed below and summarized in Figure 2.

#### 4.3.1. Initially Resectable Disease

In patients who present initially with resectable disease with favorable prognostic factors, upfront CRLM resection is the recommended treatment. However, the role of perioperative systemic therapy in these patients remains controversial. Although the addition of perioperative chemotherapy to surgical resection may eradicate micrometastatic disease, improve DFS, and facilitate patient selection by assessing tumor biology, no difference in overall survival has been found [57]. In cases where the prognostic situation is unclear, initial systemic therapy may facilitate a less extensive resection [20].

In patients with technically resectable CRLM and poor tumor prognosis, the use of perioperative treatments combined with surgical resection is considered the standard of care [20,57,88]. Combination regimens based on a fluoropyrimidine and oxaliplatin are preferred.

#### 4.3.2. Initially Unresectable Disease

Most CRLMs are initially unresectable. Although neoadjuvant systemic therapy can downstage hepatic metastases and render some unresectable cases amenable to resection (conversion therapy), the actual conversion rate remains relatively low, ranging from 5% to 30% [89]. A systematic review of 30 randomized trials published after 2003 reported a median conversion-to-resectability rate of 7.3% (interquartile range, 5–12.9%). The highest conversion rates were observed in studies employing triplet chemotherapy regimens or anti-EGFR–based therapies in patients with RAS/BRAF wild-type tumors [90].

The optimal systemic therapy regimen has not yet been established. Fluoropyrimidine-based triplets containing irinotecan and oxaliplatin (FOLFOXIRI), especially for right colon cancer liver metastases, reach higher response rates and are highly recommended when conversion therapy is required. The addition of a targeted agent should be considered based on the tumor’s molecular profile [20,88,89]. For instance, RAS mutations are negative predictive factors for the use of anti-epidermal growth factor receptor (EGFR) monoclonal antibodies (mAbs), whereas microsatellite instability (MSI-H) testing is recommended because of its predictive value in selecting patients likely to benefit from immunotherapy (first-line pembrolizumab or ipilimumab plus nivolumab [20,91]).

In patients considered for curative-intent resection of hepatic metastases, Response Evaluation Criteria in Solid Tumors (RECIST) are commonly used to determine response to preoperative therapy. These criteria define objective tumor response based on measurable changes in the sum of target lesion diameters: complete response (disappearance of all lesions), partial response (≥30% decrease), progressive disease (≥20% increase + ≥5 mm or new lesions), and stable disease (neither sufficient shrinkage nor growth). They standardize radiologic assessment across studies, with CT being the preferred method to measure lesions selected for response evaluation [92].

In these patients, resection should be carried out as soon as metastases are resectable (around 3–5 weeks after the last administration of CT, depending on the regimen used). Prolonging CT unnecessarily may lead to higher post-operative morbidity as a result of increased liver toxicity [20].

## 5. Treatment Strategies for Patients with Synchronous Colorectal Liver Metastases

The surgical approach to resecting synchronous CRLMs is complex. The treatment of patients with synchronous liver disease should be multidisciplinary and multimodal, involving different timings, sequences and types of intervention. There are three different timing options for the resection of the primary tumor and liver metastases: primary-first (colon-first), simultaneous resection or liver-first. The decision to perform a staged or simultaneous resection should be tailored to each patient’s clinical condition, tumor characteristics, and institutional experience [93,94]; ultimately, it remains an intrinsically individualized strategy. Notwithstanding, and as explained previously, whether patients should undergo preoperative chemotherapy still depends on various factors and is not strictly inherent to each strategy. At the same time, each approach is discussed in detail below. Table 2 summarizes their main characteristics.

### 5.1. Primary-First Approach

The traditional staged approach consists of resecting the primary tumor followed by adjuvant therapy, with treatment of liver metastases deferred for 3–6 months. Although it minimizes the risk of progression and complications of the primary tumor, it may allow hepatic metastases to become unresectable. Moreover, complications associated with colorectal surgery (e.g., anastomotic leakage) limit the proportion of patients who ultimately benefit from this strategy [94]. Importantly, in patients with unresectable metastatic disease and an asymptomatic primary tumor, a randomized Japanese trial demonstrated no survival benefit of upfront primary tumour resection compared with systemic chemotherapy alone, supporting a non-operative initial strategy in this setting [95]. According to data published by recent studies, this resection sequence should be limited to complicated colorectal primary tumors: perforations, where resection of the primary to remove the tumour (right colon) or suture or creating a stoma (left colon) are the recommended options, or proven obstructions, in which resection of the primary should likewise be performed first [20].

### 5.2. Simultaneous Resection

This strategy involves complete surgical resection of liver metastases at the time of primary colorectal resection. The ideal candidates for this approach are patients fit for surgery in which the primary tumor presents favorable conditions (uncomplicated) and hepatic disease is limited [96].

Because of the surgical characteristics of hepatectomies (generally performed under low central venous pressure anesthesia, Pringle maneuver, possible significant blood loss), the liver resection is usually conducted first. Once the hepatic resection is completed, the primary tumor may be removed [96]. This sequence is also usually favored as it allows inspection of the liver to rule out extensive disease, should resection of the primary tumor need to be deferred. However, considerable debate persists regarding the optimal sequence of resections and the rationale underlying each approach [97,98].

### 5.3. Liver-First Strategy

This approach was initially proposed in 2006 to allow prioritization of the most prognostically relevant disease (hepatic metastases) and facilitate subsequent integration of radiotherapy for locally advanced rectal tumors [99]. In a more recent study that comprised 7360 patients presenting synchronous CRLM, Giuliante et al. evaluated the outcome of the liver-first approach to determine patients who benefit most from this strategy. They found the liver-first approach was preferentially applied to patients with rectal primary tumors and a high hepatic tumor burden (approximately one-quarter of cases in the most recent period). As expected, this strategy was associated with shorter prehepatectomy chemotherapy duration, excellent hepatic response rates, and greater use of pelvic radiotherapy [100]. Although the liver-first strategy was initially proposed for locally advanced rectal tumors—allowing treatment of liver metastases first while providing time for pelvic radiotherapy—its use has expanded considerably in recent years. The liver-first approach may be considered in patients with CRLM who present a high hepatic tumor burden, require a rapid hepatic response, or are at risk of liver disease progression that could compromise resectability. In fact, although Giuliante et al. reported a higher proportion of patients with rectal primaries undergoing a liver-first approach, their findings, together with data from several large European registries, indicate that nearly 20–30% of cases now involve a non-rectal primary colon tumor [100,101,102]. Therefore, as highlighted throughout this manuscript, current practice favors selecting the most appropriate approach for managing CRLM based on tumor biology, hepatic tumor burden, and the anticipated optimal treatment sequence rather than focusing on exact primary tumor location.

Nevertheless, the precise role of the liver-first approach in synchronous colorectal liver metastases remains to be defined, and its oncologic superiority over alternative treatment sequences has yet to be demonstrated [93]. In fact, Sutton et al. found extrahepatic recurrence was more frequent in patients undergoing a liver-first strategy, suggesting that the unresected primary tumor resection may act as a persistent source or promoter of metastatic dissemination. Despite worse disease-free survival, overall survival remained comparable among strategies [103].

### 5.4. Two-Stage Hepatectomy

As previously described (Table 1), achieving sufficient liver volume for a well-tolerated major resection often requires a two-stage hepatectomy, among other strategies [4,65,66].

The two-stage hepatectomy aims to clear disease from the FLR during the first stage, followed by hypertrophy induction (often via portal vein embolization (PVE) or other methods) and subsequent resection of the contralateral lobe. It allows resection in patients with extensive bilobar metastases but carries a risk of progression or dropout between stages [4,65,66]. Whether resection of the primary tumor should occur during the first or the second stage depends on various factors, usually tailored to each case’s characteristics.

Other methods that are used separately or in combination with the two-stage hepatectomy to secure an adequate FLR are summarized in Table 1 and include:

PVE, which involves elective occlusion of portal venous flow to the liver segments planned for resection, redirects blood flow to the FLR and stimulates hypertrophy. It is a well-established technique with low morbidity, though tumor progression or patient dropout between stages can occur [65,66].

LVD, combining portal vein embolization with embolization of the corresponding hepatic veins to induce more robust and rapid hypertrophy of the FLR. Early studies show greater volumetric gains than PVE, but the technique is more complex and backed by limited data [104].

ALPPS merges portal vein ligation with in situ parenchymal transection, triggering extremely rapid FLR hypertrophy and enabling early completion of resection. However, this accelerated response comes at the cost of a higher rate of postoperative morbidity [4,66].

In essence, the management of CRLM seeks to achieve liver resectability while maintaining an appropriate balance between avoiding unnecessary or excessively morbid procedures and preventing hepatic disease progression that could compromise curative intent. With the expanding use of minimally invasive surgery, which has been associated with lower complication rates, this balance may be more attainable in the coming years [98,105].

## 6. Local Therapies for Colorectal Liver Metastases (CRLM)

Local therapies for colorectal liver metastases (CRLM) have undergone major consolidation over the past decade, supported by improved patient selection, technological refinement, and strengthened oncologic outcomes. Below is an updated summary of the main modalities currently used in clinical practice.

### 6.1. Radiofrequency Ablation (RFA) and Microwave Ablation (MWA)

Local ablation has evolved from an alternative option to a standard-of-care treatment for small CRLM, particularly when surgery is not feasible or when parenchymal preservation is essential. The 2023 European Society for Medical Oncology (ESMO) Clinical Practice Guidelines for metastatic colorectal cancer recognize local therapies as a potentially curative option in selected patients with oligometastatic disease or limited liver metastases, always within the context of a multidisciplinary team and experienced centers [20].

The COLLISION randomized trial [106] provided high-level evidence by evaluating both MWA alone and MWA integrated within combined treatment strategies. In its primary comparison, MWA was shown to be non-inferior to surgical resection in terms of disease-free survival for CRLM ≤ 3 cm, with significantly lower morbidity and comparable local control [106]. Importantly, the trial also reinforced the role of ablation, either as a standalone modality or as part of combined parenchyma-sparing strategies, in modern multidisciplinary management.

In addition to stand-alone ablation, the combination of surgical resection with intraoperative thermal ablation has become an established strategy for managing bilobar or multifocal CRLM while maximizing liver preservation. Early retrospective series reported inferior outcomes for combined resection–ablation compared with hepatectomy alone [107], largely due to inadequate ablative margins and older ablative technology.

However, contemporary data using modern devices have demonstrated much-improved outcomes. For example, Imai et al. [108] showed that long-term survival after combined hepatectomy plus RFA is comparable to hepatectomy alone when adequate ablative margins (>5–10 mm) are obtained. The predictable ablation zones achievable with current high-energy MWA systems, highlighted in current trials including COLLISION, further support the use of hybrid resection–ablation for deep, multiple, or anatomically challenging lesions.

Therefore, combined surgery and ablation is now considered a valuable component of parenchyma-sparing liver surgery within multidisciplinary CRLM programs, particularly for patients with multiple small lesions, bilobar disease, or limited functional reserve.

### 6.2. Irreversible Electroporation (IRE)

Irreversible electroporation (IRE) has emerged as a valuable non-thermal ablative modality for colorectal liver metastases located in anatomically complex regions, particularly when lesions lie adjacent to major vascular or biliary structures where traditional thermal techniques such as RFA or MWA are compromised by the heat-sink effect. By delivering high-voltage electrical pulses that induce apoptosis while preserving connective tissue, bile ducts, and vascular integrity, IRE enables effective treatment of tumors that would otherwise be unsuitable for thermal ablation. The pivotal COLDFIRE-2 phase II trial provided prospective evidence supporting the safety and feasibility of IRE for unresectable CRLM [109].

Current clinical practice generally reserves IRE for small metastases, typically under 3 cm, that are in direct contact with vessels larger than 3 mm, a situation in which thermal ablation often fails due to energy dissipation. The role of IRE as an adjunct to hepatectomy has also expanded, particularly in patients requiring maximal parenchymal preservation or those with a marginal future liver remnant, where complete surgical clearance would otherwise necessitate excessive loss of functional parenchyma. The relevance of IRE within multidisciplinary management is further supported by a recent systematic review and meta-analysis by Spiers et al. [110], which confirmed acceptable safety, modest local control, and a meaningful role for IRE in anatomically challenging CRLM not amenable to surgery or thermal ablation.

Despite these advantages, IRE should not be considered curative monotherapy in cases of bilobar or multifocal disease, as its local control rates remain inferior to those achieved with modern thermal ablative techniques. Nevertheless, for isolated and anatomically challenging lesions, IRE constitutes a valuable option within the multimodal treatment paradigm for CRLM.

### 6.3. Stereotactic Body Radiotherapy (SBRT)

Stereotactic body radiotherapy (SBRT) has become an increasingly relevant local treatment option for colorectal liver metastases, particularly for patients who are not candidates for surgical resection or thermal ablation due to comorbidities, lesions’ location, or insufficient hepatic reserve. SBRT delivers highly conformal, high-dose radiation over a limited number of fractions, allowing precise targeting of metastatic lesions while minimizing damage to surrounding liver parenchyma.

Over the past decade, multiple retrospective series and meta-analyses have shown that SBRT provides durable local control with an acceptable safety profile in well-selected patients. A comprehensive systematic review and meta-analysis by Petrelli et al. [111] reported 2-year local control rates of 70–90%, with low rates of grade ≥ 3 toxicity (<5%). These findings were further supported by large multicenter registry data such as the RSSearch^®^ cohort, in which Mahadevan et al. [112] demonstrated favorable control and survival outcomes for liver metastases treated with SBRT, including those of colorectal origin.

Although SBRT outcomes remain inferior to thermal ablation for small peripheral lesions, its use is particularly advantageous in tumors adjacent to vascular or biliary structures, lesions not safely accessible percutaneously, or in patients who are unable to undergo anesthesia or surgery. Technological advances—including motion management, image-guided radiotherapy, and adaptive planning—have further improved precision and reduced toxicity, reinforcing SBRT as an important organ-sparing modality within multidisciplinary management algorithms.

Despite its strengths, SBRT is typically considered a consolidative or palliative strategy rather than a curative substitute for resection or ablation. Nevertheless, for isolated, unresectable lesions or patients unfit for invasive therapies, SBRT offers a well-validated option with meaningful local control and preservation of quality of life.

### 6.4. Selective Internal Radiation Therapy (SIRT) with Yttrium-90

Selective internal radiation therapy (SIRT) with yttrium-90 (Y-90) microspheres represents an important locoregional treatment option for patients with CRLM, particularly in the setting of liver-dominant disease. By delivering high-dose β-radiation directly to tumor-bearing hepatic segments through the hepatic arterial circulation, SIRT enables targeted irradiation while sparing healthy parenchyma, making it especially valuable for patients who are not candidates for surgery or thermal ablation.

The role of SIRT in metastatic colorectal cancer has evolved substantially over the past decade. The pivotal SIRFLOX randomized trial demonstrated that adding Y-90 to first-line mFOLFOX6 (with or without bevacizumab) significantly improved liver-specific progression-free survival but did not translate into an overall survival benefit [113]. These findings were confirmed in the combined analysis of FOXFIRE, SIRFLOX, and FOXFIRE-Global, which showed improved intrahepatic control but no improvement in overall survival, leading to a more selective, individualized use of SIRT in modern practice [114].

Although no randomized trial of personalized dosimetry has been conducted specifically in CRLM, several dosimetry-driven studies have shown that higher absorbed tumor doses are associated with improved radiographic response and delayed hepatic progression. For example, voxel-based and partition-model dosimetry studies have demonstrated a clear dose–response relationship in metastatic liver disease, including cohorts with colorectal metastases [115].

In parallel, real-world evidence from large multicenter cohorts supports the use of SIRT as a salvage therapy in heavily pretreated patients with liver-dominant disease. The RESIN registry analysis, specifically evaluating metastatic colorectal cancer, demonstrated meaningful survival outcomes and acceptable toxicity after Y-90 radioembolization in a population largely refractory to systemic therapy [116].

Although SIRT is not considered curative, its capacity to induce substantial radiographic necrosis, delay intrahepatic progression, and defer the need for further systemic treatment contributes to symptom control and maintenance of quality of life in selected individuals. Advances in dosimetric planning- particularly voxel-based and personalized absorbed-dose approaches have improved patient selection and treatment safety. Consequently, Y-90 SIRT remains a key component of multimodal management for patients with liver-dominant CRLM when surgery, ablation, or other local therapies are unsuitable.

### 6.5. Hepatic Artery Infusion (HAI)

Hepatic artery infusion (HAI) chemotherapy has re-emerged as a highly active liver-directed strategy for CRLM, particularly in patients with liver-limited or liver-dominant disease. By delivering high concentrations of floxuridine (FUDR) directly into the hepatic arterial circulation through an implanted pump, HAI achieves markedly increased intrahepatic drug exposure with minimal systemic toxicity, enabling substantial tumor shrinkage, durable intrahepatic control, and, in selected cases, conversion from unresectable to resectable disease. The most compelling evidence supporting its conversion potential comes from the work of Kemeny et al., who reported that combining HAI with systemic chemotherapy resulted in conversion to complete resection in a substantial proportion of patients initially deemed unresectable, with long-term survival in the converted cohort approaching that of patients resectable at presentation [117]. These findings established HAI plus systemic therapy as one of the most effective strategies for downstaging liver-dominant metastatic disease.

Although early clinical experience with HAI was largely concentrated in high-volume centers such as Memorial Sloan Kettering Cancer Center (MSKCC), contemporary evidence demonstrates that HAI can be safely implemented beyond traditional expert institutions. In a modern cohort from a newly established HAI program, Walker et al. showed that the combination of HAI pump chemotherapy with systemic therapy achieved high hepatic response rates, favorable survival outcomes and acceptable pump-related toxicity, supporting the feasibility and reproducibility of HAI in centers without longstanding experience [118]. Together, these data reinforce the role of HAI as a powerful liver-directed modality within multidisciplinary management.

Despite these advantages, HAI remains technically complex, requiring specialized surgical expertise for pump placement, meticulous catheter management and proactive prevention of biliary toxicity. Pump malfunction, arterial complications, and biliary sclerosis remain relevant risks that limit broader dissemination, and HAI continues to be concentrated in specialized high-volume centers with dedicated multidisciplinary teams. Nevertheless, for appropriately selected patients with liver-dominant CRLM, HAI represents one of the most effective hepatic-directed treatments available, offering high response rates, opportunities for conversion to resection, and prolonged intrahepatic disease control when incorporated into modern multimodal oncologic strategies.

In addition to pump-based delivery, hepatic arterial infusion administered via implantable ports has demonstrated efficacy at least comparable to 5-fluorodeoxyuridine pump infusion, achieving high hepatic response rates and effective tumor downstaging while maintaining a favorable hepatic safety profile without increased hepatotoxicity [119].

## 7. Liver Transplantation

Liver transplantation (LT) has increasingly gained recognition as a potential treatment option for highly selected patients with unresectable CRLM, driven by refined patient selection, advances in perioperative management, and the favorable biological behavior observed in specific metastatic phenotypes. Early experiences in the 1990s yielded poor outcomes and precluded further adoption; however, the modern Oslo group revisited LT for CRLM using rigorous selection criteria and contemporary immunosuppression protocols, producing markedly improved long-term survival results. The landmark SECA-I trial demonstrated that LT in carefully selected patients with unresectable liver-only metastases, controlled primary tumor, good performance status, and responsiveness to systemic therapy resulted in a 5-year overall survival of 60%, far exceeding what is expected with systemic therapy alone [120]. Building upon these findings, the SECA-II study introduced even stricter biological criteria-lower tumour burden, sustained radiologic response, low CEA levels, and prolonged disease stability under chemotherapy-and reported 5-year overall survival rates approaching 83%, together with improved disease-free survival and reduced extrahepatic relapse compared with SECA-I [121].

Despite these encouraging results, nearly all patients experience recurrence after transplantation, most commonly in the lungs. Nonetheless, these recurrences often exhibit indolent behavior and are amenable to local therapies such as metastasectomy or ablation, and long-term survival remains favorable. Prognostic algorithms such as the Oslo score, based on CEA level, interval from primary tumor diagnosis, largest lesion diameter, and radiologic response, were defined in the Oslo experience and effectively identify patients most likely to benefit from transplantation [122].

Current international efforts, including the TRANSMET randomized controlled trial, further support the role of LT in selected CRLM, showing significant improvements in overall survival compared with chemotherapy alone [123], thereby strengthening the rationale for transplantation in biologically favorable liver-limited metastatic disease. Parallel developments in living-donor liver transplantation, machine perfusion, and expanded graft criteria continue to increase the feasibility of LT for CRLM when applied within strict selection frameworks.

Although LT for CRLM remains investigational and is constrained by organ availability, logistical considerations, and the need for highly specialized multidisciplinary coordination, contemporary evidence indicates that transplantation offers exceptional survival outcomes in a narrow but clinically relevant subset of patients with liver-only disease, favorable tumor biology, and sustained responsiveness to systemic therapy. As selection criteria evolve and prospective trials mature, LT may become an established therapeutic option for highly selected patients with unresectable CRLM within experienced transplantation centers.

In addition to international efforts, an ongoing Spanish multicentre protocol, the ELITE-CRC program, currently active at leading transplant centers, is evaluating liver transplantation for highly selected patients with unresectable, liver-only colorectal metastases using biology-driven criteria derived from the Oslo/SECA experience. This initiative represents an important step toward the structured integration of LT for CRLM within the Spanish transplantation network.

## 8. Chemotherapy-Related Issues Before Liver Surgery

Preoperative chemotherapy plays a central role in the management of CRLM, improving systemic control and enabling tumor downsizing or conversion to resectability. However, cytotoxic regimens can induce characteristic patterns of liver injury and complications that may negatively influence surgical complexity, postoperative outcomes, or intraoperative tumor detection. Understanding these chemotherapy-associated alterations is essential for optimal patient selection and perioperative planning.

Prolonged exposure to oxaliplatin-based regimens is strongly associated with sinusoidal obstruction syndrome (SOS), also known as *sinusoidal injury* or colloquially the “blue liver.” This entity results from endothelial damage, sinusoidal dilatation, perisinusoidal fibrosis, and congestion, leading to impaired venous outflow and increased intraoperative bleeding risk. SOS is reported in up to 20–70% of patients treated with oxaliplatin [124]. Severe SOS correlates with higher blood loss, increased transfusion requirements, longer operative times, and a greater risk of postoperative liver dysfunction, although its impact on long-term oncologic outcomes remains debated. The use of bevacizumab appears to reduce the incidence and severity of sinusoidal injury when administered concurrently with oxaliplatin [125].

In contrast, irinotecan-containing regimens are associated with chemotherapy-associated steatohepatitis (CASH), characterized by macrovesicular steatosis, hepatocellular ballooning, and lobular inflammation. CASH significantly increases the risk of postoperative morbidity and has been linked to higher rates of postoperative liver failure and mortality, especially when steatohepatitis exceeds 30% of the parenchyma [126]. Obesity, diabetes, and metabolic syndrome can potentiate this effect, underscoring the need for careful metabolic assessment before major hepatectomy in patients heavily exposed to irinotecan.

Another clinically relevant consequence of preoperative chemotherapy is the phenomenon of “missing metastases,” defined as lesions that are radiologically evident before chemotherapy but undetectable intraoperatively, despite close evaluation. These disappearing CRLM occur in up to 20–30% of patients receiving modern conversion therapy [127]. Although some lesions truly undergo complete pathological response, up to 80% harbor residual microscopic disease and therefore carry a substantial risk of local recurrence if not removed or ablated. High-quality preoperative imaging, intraoperative ultrasound, and meticulous correlation with baseline scans are essential to minimize the risk of leaving untreated viable disease.

In addition to parenchymal injury and disappearing lesions, preoperative chemotherapy may influence postoperative complication rates. SOS is associated with increased perioperative morbidity, while CASH is linked to worse short-term survival after major hepatectomy. Highly intensive regimens such as FOLFOXIRI may further amplify toxicity if continued for extended durations. Accordingly, most guidelines recommend limiting preoperative chemotherapy to no more than 6 cycles in patients already deemed resectable and avoiding prolonged therapy when major hepatectomy is anticipated.

Together, these chemotherapy-related issues highlight the importance of balancing oncologic benefit with hepatic safety. Multidisciplinary coordination, careful timing of surgery, assessment of liver parenchymal quality, and integration of hepatobiliary surgical judgment remain essential to mitigate risks and optimize outcomes in patients undergoing liver resection after systemic therapy.

## 9. Conclusions

The management of colorectal liver metastases has evolved into a dynamic, biology-driven and multidisciplinary process, in which treatment strategies are continuously adapted according to disease behavior, patient condition and treatment response. While surgical resection remains the cornerstone of curative intent, it should no longer be considered an isolated intervention, but rather one integrated component within a broad and evolving therapeutic arsenal.

Modern outcomes rely on the central role of multidisciplinary team decision-making, which enables accurate assessment of resectability, appropriate patient selection, and optimal sequencing of systemic, locoregional and surgical treatments. Advances in imaging, liver functional assessment, parenchyma-sparing surgery and liver-directed therapies have expanded resectability boundaries while maintaining safety and oncologic rigor.

Ultimately, an MDT-centric strategy that incorporates tumor biology alongside technical and clinical factors is essential to maximize curative opportunities and improve long-term survival in patients with colorectal liver metastases.

## Figures and Tables

**Table 1:** Main approaches for optimizing FLR.

Strategy	Principle	Advantages/Limitations
Portal vein embolization (PVE)	Elective obliteration of portal blood flow to a selected portion of the liver	Well established, low morbidity/Progression or dropout between stages
Liver venous deprivation (LVD)	Simultaneous embolization of portal and hepatic veins of resected lobe	Greater and faster hypertrophy/Technically demanding; limited data
Associating Liver Partition and Portal vein ligation for Staged Hepatectomy (ALPPS)	Combines portal vein ligation with parenchymal transection to trigger rapid hypertrophy	Allows early completion of resection/Higher rate of complications
Two-stage hepatectomy	Initially clear FLR lesions, then contralateral lobe after hypertrophy	Enables bilobar disease resectability/Progression or dropout between stages

FLR: Future Liver Remnant; PVE: Portal Vein Embolization; LVD: Liver Venous Deprivation; ALPPS: Associating Liver Partition and Portal Vein Ligation for Staged Hepatectomy.

**Table 2:** Comparison of synchronous CRLM treatment strategies.

Approach	Advantages	Disadvantages	Ideal Patient
Primary-first	− Reduces risk of primary-site complications.	− Delay in liver metastasis treatment may allow progression of hepatic disease (prognosis). − Primary surgery complications may delay liver surgery.	− Certain symptomatic primaries.
Simultaneous	− Efficient and early treatment of all disease with only one operation requiring general anesthesia.	− Prolonged operative time and potentially increased risk of perioperative morbidity.	− Low liver tumor burden and “easy” resectable colon cancer.
Liver-first	− Prioritizes the site which often drives prognosis. − Can exploit the window while the primary (especially rectal) is being addressed by neoadjuvant therapy or awaiting surgery.	− Risk of primary tumor becoming symptomatic. − Liver surgery complications may delay primary surgery.	− Rectal primary tumor and a high number of CRLMs resected.

CRLM: Colorectal Liver Metastases.

## Data Availability

Data sharing is not applicable since no new data has been generated.
